# Temporal resolution shapes inferred higher-order interactions and information representation in neural population activity: A data-driven approach using minimally complex models

**DOI:** 10.1371/journal.pone.0322347

**Published:** 2026-10-26

**Authors:** Fariba Jangjoo

**Affiliations:** Kavli Institute for Systems Neuroscience and Centre for Algorithms in the Cortex, Faculty of Medicine and Health Sciences, Norwegian University of Science and Technology, Trondheim, Norway; Shanghai Jiao Tong University, CHINA

## Abstract

Studying limited datasets derived from complex interacting systems, such as neural population activity, represents an important and challenging task. The level of detail in recorded activities is highly influenced by the temporal resolution at which the activities are studied. However, does more detail always lead to a correct assessment of the system? This study examines how temporal resolution influences the inference of higher-order statistical dependencies and information representation in population-level neuronal activity. Using a minimally biased data-driven statistical inference framework, minimally complex models, we studied grid cell populations in the medial entorhinal cortex across a broad range of temporal resolutions. Results based on 23 grid cell recordings including different animals and behavioral states show that at an intermediate temporal resolution ($𝒪(10^{2})$  ms), the inferred model captures significant higher order dependencies alongside efficient information representations, whereas the finest and the coarsest temporal resolutions lead to poor information representation. The informative temporal resolution can be identified by two criteria: the bin size at which the inferred interaction structure is most consistent across population sizes, and the peak of the model's information representations, which agree closely across recordings. These findings indicate that temporal resolution is a critical factor in the inference of higher order neural structure and highlight that resolution-aware modeling is crucial for accurate analysis and characterization of collective dynamics in complex neuronal systems.

## Introduction

The amount of information retained from neural activity depends strongly on the temporal resolution used during recording or analysis. This factor can significantly impact the interpretations of neural dynamics. Fine temporal resolution is able to capture detailed and rapid neural dynamics, whereas coarse resolutions may smooth out dynamical patterns, leading to information loss [1–4]. Consequently, the dynamical pattern representation influences the inference of interactions based on population activity data. While numerous studies have investigated interactions in complex neural systems, the influence of time resolution, particularly on higher-order interactions, has not been systematically addressed. Time resolution can significantly influence the inference of the interactions and subsequently how we interpret the complex interacting systems.

Understanding interactions based on data is one of the gateways to understand complex interacting systems such as neural networks. This gives us insight into how information is encoded and processed. Traditional studies have focused on models with pairwise interactions, which capture the correlations between two elements. However, making prior assumptions about the underlying interaction structure is a biased approach when dealing with complex systems. By assuming pairwise interactions, deeper structures within interacting systems where higher-order interactions simultaneous correlations among multiple elements are not captured. Higher-order interactions offer a more informative understanding of neural dynamics and functional connectivity [5,6]. In addition, consequences such as a compact form of encoding of information and processing are not effectively explained by simpler models such as pairwise models [7,8]. Studies indicate that pairwise models inadequately represent complex systems, as these fail to capture the rich dynamics of higher-order interactions in large neural populations [5,7,9]. For instance, Montani et al. showed that interactions beyond pairwise, are essential in order to capture the complexity of the neural responses in the somatosensory cortex, where pairwise models alone are not able to properly approximate the information carried by the population of neurons [10]. Similarly, Zylberberg and Shea-Brown observed that non-linearity in input could lead to higher-order correlations, improving information transmission in neural populations [11]. Other studies confirmed that even within local cortical and retinal networks, higher-order correlations reveal deeper structure which is not captured by pairwise interactions alone, especially as population size grows [8,12]. In addition, studies showed the necessity of the higher-order interactions in understanding widespread distributions of neural activity [13,14]. In practical applications, such as visual processing or in general when it comes to modeling biological systems studies highlight the importance of incorporating higher-order interactions to accurately model neural population activity [5,7,8,15–17].

Cortical activity has been linked to various covariates such as brain states [18]. Studies show that higher-order interactions are more prominent during specific conditions, such as heightened synchrony or attention. For example, research has demonstrated that the correlation structure of neural networks is preserved across behavioral states, including sleep and active waking behavior [19,20], while other papers show that higher-order interactions in the brain are present during the resting state [21,22]. Correlations among neurons also determine whether a population code is synergistic or redundant [23]. In the visual cortex, research demonstrates that during active perception or directed attention to a stimulus, higher-order correlations can enhance the brain's ability to discriminate between visual features [24]. This enhanced discrimination may arise from the brain's use of higher-order interactions to create distinct representations of stimuli, thereby increasing the efficiency of encoding and processing information. Similarly, in sensory systems, higher-order correlations become more pronounced during states of rich sensory input or heightened perceptual engagement. For example, Ohiorhenuan et al. observed that local cortical firing patterns show high-order correlations that are flexibly and rapidly reorganized by visual input [8]. This suggests that higher-order interactions may be critical for encoding and processing information during complex sensory experiences.

This might lead to the assumption that higher-order interactions are inherently tied to specific states or regions without adjusting for temporal factors [25]. Representing information at fine temporal resolution may mask true dependencies by emphasizing stochastic fluctuations, whereas coarse resolutions may introduce spurious correlations. Therefore, the chosen temporal resolution might not capture the accurate picture and consequently does not provide correct insights. By incorporating the fact that information content in neural spike trains is highly affected by the temporal resolution at which the system is studied [26,27], one can present the data by a more efficient representation of information which explains the data with reliable functional dependencies among elements.

Functional dependencies among elements in a complex system can range from pairwise interactions to higher orders of interaction, and the inference approach must be able to overcome computational limitations when dependencies are complicated. There have been attempts at inferring this type of interactions. Traditionally, approaches such as maximum entropy models that attempt to account for higher-order interactions struggled with computational demands as the number of interacting elements grows [10,28–30]. Moreover, these models rely on predefined assumptions about the significance of interaction orders, which can restrict their compatibility when it comes to explaining complex systems based on limited data [7,31].

This study shows the critical role of temporal resolution in understanding the underlying complex system. Using grid-cell recordings from the medial entorhinal cortex, inference of the interaction structure is examined across a broad range of temporal resolutions employing minimally complex models [32]. Unlike conventional approaches, this model is able to reveal which orders of interaction are most relevant for a given data set without predefined structural assumptions, allowing the detection of isolated neurons, pairwise interactions and higher order dependencies. Using this approach we examine extensive datasets considering various neuronal geometries (which arise from grid spacing), in internal state (e.g., sleep), and in experimental configuration. Across different conditions, it reveals how higher-order correlations emerge differently across temporal resolutions, underscoring that informational content and interaction significance are inherently shaped by temporal framing.

This study introduces a systematic approach for quantifying how temporal resolution shapes the inference of higher order interactions in complex systems. By evaluating model evidence, component structure, and interaction order across a wide range of temporal resolutions, the approach identifies a narrow region of temporal resolution, at which the encoding complexity is minimal and at the same time almost consistent across different population sizes, and information content is maximal, where higher-order interactions play a dominant role. This region is identified where the two independently defined criteria $b_{\sigma (b)}^{*}$ and $b_{\tilde{\ell }}^{*}$ (Materials and methods) are jointly satisfied. Overall, this study emphasizes that temporal resolution is not merely a pre-processing parameter, but a critical analytical factor that must be systematically evaluated to ensure reliable and less biased interpretations. The approach in this paper provides a general methodological framework for determining the most informative temporal scale for a given data set, with potential applications extending beyond neuroscience.

## Materials and methods

### Inference framework for binary data from complex interacting systems

In order to study the interactions defined by a binary data set recorded from a complex interacting system, we employ the framework of minimally complex models (MCMs) [32]. This framework is developed for binary data where the response of each system variable is simplified into two states. Given a sequence of binary data set, the temporal resolution is defined as the bin size that summarizes the response of the system variable. For simplicity, if the system variable is responsive somewhere in a particular bin with a specific size (The method of determining the activity of the variables is defined in Binning procedure), the response of the variable is summarized as active. On the other hand, if the variable is non-responsive in that particular bin, the response of the variable is defined as silent.

For a system with *n* number of variables, a single observation of the system's state can be defined as $S=\left(s_{1},s_{2},...,s_{n}\right)$, where each $s_{i}$ represents the binary state of variable *i*. Therefore, a set of *N* independent observations of system state can be expressed as $𝒮=\left(S^{\left(1\right)},...,S^{\left(N\right)}\right)$. Given a system state S, one relevant question to ask is about the underlying structure of the binary data of the system's state, which defines the connectivity patterns among the variables in the system. Classically, in the realm of statistical inference, the underlying model from which the data is generated is usually predefined. If we assume that the variables are connected two by two (pairwise interaction), the suggested underlying model could be the pairwise Ising model. However, one can object to this assumption and suggest higher order interactions in the system as we do not have any precise information on the generative model. Therefore, in order to find the best structure for a given binary data set, one must evaluate all the possible structures that can exist for a system with *n* number of variables. However, evaluating all possible structures is an infeasible computational task. Therefore, according to the framework suggested by C. de Mulatier et al. [32], we restrict our search to a particular family of models called MCMs. According to this framework, the inferred structure has minimal information-theoretic complexity while maximizing the model's evidence as a measure of how well the model describes the data. If each particular structure is defined as a model, by comparing the evidence of models (probability of the data given the model, integrated over its parameter values), one can find the best structure as the model with the largest evidence.

The MCM framework assumes that a system configuration S is drawn from an exponential family distribution. In this framework, each variable $s_{i}$ is binary (taking values of ±1), and the probability of observing S is given by;


$$\begin{array}{c} p(S|g,ℳ)=\frac{1}{Z_{ℳ}(g)}\exp\left(\sum_{\mu \in ℳ}g^{\mu }\phi^{\mu }(S)\right), \end{array}$$

where ℳ represents the set of operators encoding interactions of arbitrary order, with each interaction defined by the function $\phi^{\mu }(S)$. Each operator μ indicates which variables participate in an interaction, and can be represented as a binary vector with length *n*, or equivalently by a binary integer of *n* bits. For instance in a system with *n* number of spin variables, if a spin operator is product of three variables *s*_1_, *s*_4_ and *s*_5_, then the binary code (μ) of this particular spin operator is 000011001 or the integer representation $2^{0}+2^{3}+2^{4}=25$. Furthermore, $g^{\mu }$ denotes the interaction strength for operator μ, and $Z_{ℳ}(g)$ is the partition function ensuring normalization of p(S∣g,ℳ).

In absence of any prior knowledge, one must evaluate all the possible models generated by *n* number of variables ($2^{2^{n}-1}$ number of models), which is an infeasible task. In order to tackle the computational challenge of searching among all the models, MCMs framework constrain the search such that the best models are defined by orthogonal subsets called Independent Complete Components (ICCs).

Each ICC (denoted as ${ℳ}_{a}$) is constructed from a set of $r_{a}$ independent operators, which cannot be obtained by multiplying other existing operators in the set. The operators within each ICC are built on a basis set. If the basis set consists of individual binary variables, then the basis would include only those variables without any higher-order products. On the other hand, the basis set can be changed to include independent combinations that capture maximal bias. Each complete component includes all $2^{r_{a}}-1$ operators that are generated by its $r_{a}$ basis operators, and consequently the number of operators for the entire model is defined as $\sum_{a\in 𝒜}(2^{r_{a}}-1)$. The combined basis set for the model ℳ can therefore be obtained as the union of the bases $\beta_{a}$ for each ${ℳ}_{a}$;


$$\begin{array}{c} \beta =\cup_{a\in 𝒜}\beta_{a}. \end{array}$$

Each ICC ${ℳ}_{a}$ is orthogonal to others, which means that the interactions within each component are independent of others: ${ℳ}_{a}\cap {ℳ}_{a^{\prime }}=\emptyset$ for $a\neq a^{\prime }$.

Based on Bayesian model selection, the best model is the one that maximizes the posterior probability P(ℳ∣𝒮). Using Bayes theorem $P(ℳ|𝒮)\propto P(𝒮|ℳ)P_{0}(ℳ)$, one can attempt to calculate the evidence as


$$\begin{array}{c} P(𝒮|ℳ)=\int_{{\mathbb{R}}^{M}}dg\prod_{i=1}^{N}p(S^{\left(i\right)}|g,ℳ)p_{0}(g|ℳ), \end{array}$$

where $p_{0}(g|ℳ)$ is the prior distribution over parameters.

If we define $k_{\beta_{a}}$ as the count of basis operator $\beta_{a}$ in the data set 𝒮 with length *N*, then the model evidence for a model MCM is calculated as [32]:


$$\begin{array}{c} P(𝒮|ℳ)=\frac{1}{2^{N(n-r)}}\prod_{a\in 𝒜}\frac{\Gamma \left(2^{r_{a}-1}\right)}{\Gamma \left(N+2^{r_{a}-1}\right)}\prod_{\beta_{a}}\frac{\Gamma \left(k_{\beta_{a}}+\frac{1}{2}\right)}{\sqrt{\pi }}, \end{array}$$

where Γ denotes the Gamma function. This formulation of the evidence enables comparison across MCMs, leading us to selection of the model with the highest evidence $\hat{ℳ}$ which is the best fit for the data set.

Consequently, the maximum likelihood distribution will take the following simple format;


$$\begin{array}{c} P(𝒮|\hat{g},ℳ)=\frac{1}{2^{N(n-r)}}\prod_{a\in 𝒜}\prod_{\beta_{a}}{\left(\frac{k_{\beta_{a}}}{N}\right)}^{k_{\beta_{a}}}. \end{array}$$

Here $\hat{g}$ denotes the maximum likelihood estimate of parameter g obtained from the data. This formula simplifies the exhaustive calculation of the log-evidence, while avoiding over-fitting.

Through MCMs framework, the algorithm iteratively explores through the space of possible models, updating to models that lead to increase of the model evidence, until no alternative model yields to higher model evidence. Therefore, the final model $\hat{ℳ}$ will provide an optimized description based on the data set. The MCMs algorithm can be summarized in two steps; (1) Searching for the best basis, and (2) Obtaining the optimal MCM through hierarchical merging.

**Step 1: Heuristic search for the best basis**

Input: binary data set 𝒮, maximum interaction order *k*

Output: best basis β

1. Initialize basis β with single variable operators (β = *s*).

2. Generate all candidate operators up to order *k* from basis β.

3. Among all candidates, identify a set $\beta^{\prime }$ of *n* independent operators that are maximally biased.

4. $\beta \leftarrow \beta^{\prime }$.

5. Repeat 2-4 until $\beta^{\prime }$ = β.

6. Return β.

**Step 2: Hierarchical merging to obtain the optimal MCM**

Input: best basis β from step 1

Output: optimal MCM

1. Construct the independent model (IM) defined by basis β from step 1. This corresponds to an MCM with *n* ICCs of rank 1.

2. For all pairs of ICCs, compute the evidence of the model obtained by merging the pair.

3. Identify the merge that yields the maximal increase in evidence.

4. If such a merge exists, merge the corresponding ICCs.

5. Repeat 2–4 until no merge increases the evidence.

6. Return the final MCM with maximal evidence along the hierarchical merging trajectory.

In this paper, the MCMs framework is presented and utilized without any modification, and all the mathematical formulation and algorithms are aligned with the initial framework suggested by C. de Mulatier et al. [32]. This inference framework is utilized in order to infer the underlying structure within the datasets considered in this study. The metrics from inferred models such as ICCs, log-evidence and the structure of components/operators, are used for further analysis.

### Binning procedure

In order to evaluate the impact of the binning method on the results, two different binning methods are considered. Generally, binning involves dividing the entire duration of recorded data *T*, into *N* bins, where *N* is determined by dividing the recorded length by the chosen bin size ($N=\frac{T}{\text{bin size}}$). Thereafter, to summarize each bin's data into a single representative value, we approached it using two different methods:

- **Presence of Spikes Method:** In this method, we assign a value of 1 to a bin if there is at least one instance of activity (i.e., the neuron fired) within that bin; otherwise, the bin is denoted as 0 to indicate inactivity.
- **Average Firing Rate Method:** In this approach, we compute the mean firing rate within each bin *R*_bin_, and compare it to the mean firing rate over the entire recording $R_{T}$. If $R_{\text{bin}}\geq R_{T}$, the bin is marked as active (1); otherwise, it is marked as inactive (0). Since this threshold is defined based on the recording's own mean firing rate, rather than a fixed value, it adapts to the baseline activity level for each data set.

### Random realizations

In order to have better statistical evaluation, we generate random realizations of the recorded binary data set. This procedure starts by specifying a fixed duration *T* for all realizations. Given this duration, we extract multiple sub-datasets (or “realizations”) of length *T* from the original data set, which can be regarded as independent samples for further analysis. The analysis is done for various population sizes of cells *n*, where *n* is randomly chosen from the total cell population in the data set. For each chosen cell population size *n*, we draw 200 independent realizations of the recorded dataset, each of length *T*, by randomly sampling subsets of cells from the entire data set.

Each realization is then binned according to the defined binning method, using a defined bin size, which summarizes the activity within each bin. Thereafter, quantities of interest are calculated for these binned realizations at varying bin sizes.

In order to account for potential statistical biases due to limited data, we also evaluate each analysis on the shuffled version of each realization, where before binning, the sequence of spikes for each cell is randomly permuted. Thereafter, the binning is done over the shuffled version of each realization. This shuffling is intended to remove any inherent temporal correlations while preserving the original firing rates. The results of the shuffled version of the realizations can be used to remove the bias due to limited data sampling. In order to do so, we evaluated the “difference” between the results of the shuffled and un-shuffled versions. By examining the difference, we aim to isolate the effects of temporal correlations and other structured dependencies present in the original data set, from random noise or baseline levels in the shuffled data. In figures, results from the original (un-shuffled) data are denoted with the subscript *O* (Original), while results from the shuffled data are denoted with *S* (Shuffled).

### Distribution of active cells across different bin sizes

#### Active cell fraction heatmap.

After binning each random realization into a specific bin size, one can investigate the number of bins that have a specific fraction of active cells, or in other words, the probability of observing a fraction of active cells considering *N* data points. Clearly, in this procedure, when the bin size value is the finest (i.e., 1 *ms*), each cell can be active at most once within a bin. While considering the greater values of bin sizes leads to coverage of a longer time span, which might lead to coverage of multiple activities of one cell. In this analysis, we are not concerned about the number of spikes that each cell emits within each bin, rather the presence of activity of each cell. After counting the present cells in each bin, one can obtain the distribution of each fraction. In order to do so, the fraction of the bins with the occurrence of each active cell fraction is counted (an illustrative example is provided in Table 1). The distribution can be observed in a heat-map, where the y-axis shows the active cell fraction ($n_{A}/n$), the x-axis shows the value of the bin size, and the heat color shows the probability of observing $n_{A}/n$ having *N* data points defined by the bin size. This probability is evaluated over 200 different realizations of the data with a length of 10 minutes.

**Table 1:** Visual example of active cell fraction and its distribution. Demo spike trains for three cells are shown across several time bins. For each time window, the fraction of active cells is obtained as the number of cells with spike (value 1) divided by number of cells. Thereafter, the distribution of these fractions is obtained by counting the occurrence of each fraction divided by total number of bins.

**Cell 1**	0	1	0	1	0	0	1
**Cell 2**	1	0	0	1	0	1	0
**Cell 3**	0	0	1	0	1	0	1
**Active cell fraction ($n_{A}/n$)**	1/3	1/3	1/3	2/3	1/3	1/3	2/3
**Distribution $p(n_{A}/n)$**	*p*(1/3): 5/7, *p*(2/3): 2/7						

#### Mean active cell fraction and logistic fit.

For each bin size *b*, we summarized the population-level distribution of active cell counts $P(n_{A}=j|b)$ by its expectation value;


$$\begin{array}{c} \left\langle \frac{n_{A}}{n}\right\rangle (b)=\sum_{j=0}^{n}\frac{j}{n}\;P(n_{A}=j|b), \end{array}$$

Across all the recordings and binning methods considered in this paper, curves $y_{n}(b)$ (the mean active cell fraction at bin size b for population size n) for different values of population sizes agree closely with each other at every considered bin size,


$$\begin{array}{c} {median}_{n,b}|y_{n}(b)-\langle n_{A}/n\rangle (bbig|=0.0005,\;\max_{n,b}|y_{n}(b)-\langle n_{A}/n\rangle (b)|=0.016, \end{array}$$

therefore their median across *n*, $\langle n_{A}/n\rangle (b)$, provides a faithful representative summary curve for each individual population size. With this median curve established, a four parameter logistic function can be fit to this median curve as a function of log(b), yielding the sigmoid fit curve.

#### Divergence bin $b_{div}^{*}$.

In order to compare how the considered binning methods throughout this paper diverge as bin size increases, we evaluate the divergence bin $b_{div}^{*}$ as the smallest bin size at which the two binning methods mean active cell fraction curves differ by at least 5 percentage points (S5 Fig);


$$\begin{array}{c} b_{div}^{*}\;=\;\min\left\{\;b\;:\;\left|{\left\langle \frac{n_{A}}{n}\right\rangle }_{AvgFR}\;(b)-{\left\langle \frac{n_{A}}{n}\right\rangle }_{Presence}\;(b)\right|\geq 0.05\;\right\}, \end{array}$$

### Independent complete components and logevidence

After finding the best model (according to MCMs steps) for each random realization at different bin sizes, we can perform analysis on the ICCs and the Log-evidence inferred from the inference framework. Having the ICCs and log-evidence of the inferred model, we investigate two main quantities; the normalized number of ICCs ($\left\|k_{\beta_{a}}\neq 0\right\|$) where this value is normalized by the number of cells *n*, and the log-evidence of the detected model (log(P(S|ℳ))) per data point *N* both normalized by *n* and un-normalized format. All these values are studied on average with their standard deviation, where these statistics are calculated considering all the random realizations. Bear in mind that the number of independent complete components is calculated by counting basis $\beta_{a}$ for which $k_{\beta_{a}}\neq 0$. By normalizing the values to the number of cells *n*, we can obtain a better insight when comparing results of realizations with different values of *n*.

### Orders of interaction

Another interesting attribute to evaluate is the order or equivalently the size of detected components and associated operators of the best model that describes the data. When observing the order of interactions, we need to perform two distinct procedures for components and operators. In order to evaluate the size of the components, the number of unique cells is considered, where this set of cells is obtained from the union of all the cells presented in all operators of the corresponding component. However, when dealing with operators, the size of all the operators of each ICC is evaluated.

Thereafter, in order to calculate the probabilities, we can investigate the number of components/operators with order *i* in which the components/operators contain *i* number of cells. In order to examine how the fraction of components of order *i* changes with bin size, the fraction of components with order *i* within a model is plotted, averaged over all the considered realizations, for each bin size. However, the plots are not visually clear due to small values. In order to have visually clear plots, a cube root transformation was applied to the values. This transformation re-scaled the values by raising them to the power of $\frac{1}{3}$, which reduces the impact of large outliers while retaining the relative differences between values. In heat-maps, the color bars show the respective original values, while the y-axis values in curve plots are presented in cube root transformation.

### Cross-recording consistency criteria

#### ICC/n crossover bin $b_{\sigma (b)}^{*}$.

For simplicity, the normalized ICC count difference at bin size *b* and population size *n* is renamed as $c_{n}(b)$;


$$\begin{array}{c} c_{n}(b)\;\equiv \;\frac{\langle \|k_{\beta_{a}}\neq 0\|\rangle_{O}-\langle \|k_{\beta_{a}}\neq 0\|\rangle_{S}}{n} \end{array}$$

In which the $\langle \cdot \rangle_{O}$/$\langle \cdot \rangle_{S}$ throughout this paper are calculated over random independent realizations from a fixed data set. Considering the $c_{n}(b)$ plots for different population sizes *n*, the cross-*n* median curve is defined as $\tilde{c}(b)={median}_{n}\;c_{n}(b)$, along with its dispersion as $\sigma (b)={std}_{n}\;c_{n}(b)$. Note that since $c_{n}(b)$ is defined as the difference between original and shuffled case, we expect that the value of $c_{n}(b)$ collapses toward zero for very large bin sizes, where the dispersion is naturally small. This happens as a result of limited data which creates bias at coarse temporal resolution. Therefore, we restrict our attention to the bin sizes that are meaningful. If we define $b_{\min}=\text{arg}\min_{b}\tilde{c}(b)$ as the bin size at which the median curve is minimized, we can define a meaningful range around this minimum;


$$\begin{array}{c} ℬ=\{\;b\;:\;|idx(b)-idx(b_{\min})|\leq 5\;\},\;b_{\min}=*{arg\;min}_{b}\tilde{c}(b), \end{array}$$

where idx(*b*) defines the index of the bin size in the ordered list of bin sizes. This range spans up to five bin sizes on each side of the minimum. The choice of window size 5 for this data set is such that it spans the range over which the plots for different population sizes *n* visually converge, and it is checked against the individual recordings; the corresponding plots are available at the public repository (see Data Availability Statement). The ICC/n crossover bin is then defined as the following;


$$\begin{array}{c} b_{\sigma (b)}^{*}=*{arg\;min}_{b\in ℬ}\;\sigma (b). \end{array}$$

This is the bin size at which the plots for different values of *n* have the minimal distance to each other within the restricted range ℬ.

#### LogEv/n peak bin $b_{\tilde{\ell }}^{*}$.

For simplicity, we rename the normalized log-evidence difference at bin size *b* and population size *n* as the following;


$$\begin{array}{c} \ell_{n}(b;\equiv \;\frac{\langle \log(P(S|M))\rangle_{O}-\langle \log(P(S|M))\rangle_{S}}{N\times n} \end{array}$$

Then the bin size at which the maximum of the median plot $\tilde{\ell }(b)$ ($\tilde{\ell }(b)={median}_{n}\;\ell_{n}(b)$) takes place, is defined as $b_{\tilde{\ell }}^{*}=*{arg\;max}_{b}\;\tilde{\ell }(b)$.

#### Informative bin size $b_{info}^{*}$.

It should be emphasized that, for convenience, $b_{\sigma (b)}^{*}$ and $b_{\tilde{\ell }}^{*}$ are reported as single bin sizes. However, each is chosen as the best point within a broader range of bin sizes that perform nearly as well, since $\tilde{c}(b)$ and $\tilde{\ell }(b)$ change smoothly with *b*. Therefore, the informative bin size is better understood as a narrow region where both criteria are approximately jointly satisfied, with $b_{\sigma (b)}^{*}$ and $b_{\tilde{\ell }}^{*}$ as representative points within this region, rather than as an exact, uniquely determined value. Throughout this study, we define the informative bin size $b_{info}^{*}$ as the geometric mean of the two mentioned criteria, rounded (in log space) to the nearest bin size evaluated in this study;


$$\begin{array}{c} b_{info}^{*}\;\equiv \;\mathrm{round}\;\left(\sqrt{b_{\sigma (b)}^{*}\cdot b_{\tilde{\ell }}^{*}}\right), \end{array}$$

therefore, the region can be represented by a single value $b_{info}^{*}$.

#### Agreement between criteria.

The scatter plot of the joint value $\left(b_{\sigma (b)}^{*},b_{\tilde{\ell }}^{*}\right)$, can show if for each recording, two representative bins represent the same value, where the line $b_{\sigma (b)}^{*}=b_{\tilde{\ell }}^{*}$ marks the exact agreement between two points.

Another way to evaluate the distance between two representative bin sizes in scatter plot is to define $\log_{2}$ fold-change as the following;


$$\begin{array}{c} \Delta_{i}=\log_{2}\;\left(\frac{b_{\tilde{\ell },i}^{*}}{b_{\sigma (b),i}^{*}}\right), \end{array}$$

where *i* denotes each recording. The $\left\{\Delta_{i}\right\}$ therefore quantifies the log-distance from the diagonal line $b_{\sigma (b)}^{*}=b_{\tilde{\ell }}^{*}$. Given these values, the histogram of $\left\{\Delta_{i}\right\}$ indicates the proportion of recordings at each distance value. The choice of $\log_{2}$ ratio rather than a simple $b_{\tilde{\ell },i}^{*}-b_{\sigma (b),i}^{*}$ has two reasons: since the bin sizes used throughout this paper are generally powers of 2, $\log_{2}$ ratio can count “grid steps” between the two bin sizes, regardless of their absolute magnitude. Also, $\Delta_{i}$ is a symmetric scale-free parameter that assigns the same weight to the points that are equidistant from the exact agreement line (marked by $\Delta_{i}=0$) on both sides. By this definition, the percentage of recordings whose $\log_{2}$ ratio is within a factor of two distance with respect to the exact agreement line is defined as the fraction of recordings which satisfy $|\Delta_{i}|\leq 1$.

### Dataset

In this study, the neural data used for analysis were recorded from grid cells [33] in the medial entorhinal cortex by Gardner et al. [34]. Data acquisition with Neuropixels silicon probes allowed for simultaneous recording of neural activity from multiple grid cells over a long period, which is essential for our type of analysis. We restricted our analysis to two rats named as R and Q, for which the data from rat R is recorded over two different days. In general, this recording includes sessions across various tasks, behavioral states, and cellular modules, which gives a richer understanding of dependencies on possible covariates.

The recording is explained in more detail based on behavioral states which are indicated by task and state of the animal (e.g., during sleep), and modules which represent groups of grid cells that exhibit similar spatial scales and orientations in their firing patterns. Behavioral state or type of experiments are categorized as:

- **Open Field (OF):** A square-shaped environment where the rats could freely explore for randomly scattered food crumbs. Recordings were made to capture the hexagonal firing patterns of grid cells during active navigation.
- **Wagon Wheel (WW):** A circular track with radial arms, resembling a wagon wheel. This environment provides a structured spatial setting to study how grid cells adapt to a different spatial layout.
- **Rapid Eye Movement (REM) and Slow-Wave Sleep (SWS):** Recordings were performed to capture grid cell activity in two different sleep phases.

Each module's spatial configuration is characterized by a different level of grid spacing. Rat R was recorded across three modules, in which the spacing between peaks in the hexagonal grid pattern increases progressively from module 1 to module 3. For rat Q, two modules are considered in which module one features closer grid spacing while the second one covers a wider spacing. In this paper, the session's recording is denoted as “Rat/Module/Experiment/Day”. In this setting, day one is encoded as I and day two as II. In the case that there is no specified day, the day label is absent. For instance, R1WWI is the recording from the first module of the rat R in WW experiment, which is recorded on day 1. Details of each recording are presented in Table 2.

**Table 2:** Information on the Grid cell’s recording of rat R and Q in 1 millisecond temporal resolution. This table summarizes the sessions, listing the rat name, Module ID, Experiment, the day of the recording, number of recorded cells and the total duration of each session at 1 millisecond temporal resolution.

Rat	Module ID	Experiment	Day	ID	Number of Cells	End (ms)
R	1	OF	I	R1OFI	166	8476000
	2	OF	I	R2OFI	168	8476000
	3	OF	I	R3OFI	149	8476000
	1	WW	I	R1WWI	166	3622000
	2	WW	I	R2WWI	168	3622000
	3	WW	I	R3WWI	149	3622000
	1	OF	II	R1OFII	189	2387001
	2	OF	II	R2OFII	172	2387001
	3	OF	II	R3OFII	183	2387001
	1	REM	II	R1REMII	189	1422016
	2	REM	II	R2REMII	172	1422016
	3	REM	II	R3REMII	183	1422016
	1	SWS	II	R1SWSII	189	6185908
	2	SWS	II	R2SWSII	172	6185908
	3	SWS	II	R3SWSII	183	6185908
Q	1	OF	_	Q1OF	97	3397001
	2	OF	_	Q2OF	66	3397001
	1	WW	_	Q1WW	97	6378001
	2	WW	_	Q2WW	66	6378001
	1	SWS	_	Q1SWS	97	5967956
	2	SWS	_	Q2SWS	66	5967956
	1	REM	_	Q1REM	97	1052066
	2	REM	_	Q2REM	66	1052066

## Results

In this study, the results of the analysis on 23 distinct recordings of grid cells in the medial entorhinal cortex of two rats (R and Q) across different experimental states, days and modules are extensively evaluated (Information of the recordings is presented in Table 2). From each recording, 200 random realizations with duration *T* (*T* = 10 minutes) and *n* number of cells are drawn independently. Thereafter, three types of analysis are performed: analysis on the original (un-shuffled) realizations, denoted by *O*; analysis on the shuffled realizations, denoted by *S*; and the difference between the two types in order to understand the results by avoiding the statistical biases that come from limited data. Thereafter, in order to understand the effect of the temporal resolution on our analysis, each individual realization's data (Original or Shuffled) is evaluated for different values of bin sizes ranging from the finest resolution *b* = 2^0^ = 1 ms to the coarsest considered resolution in this study *b* = 2^15^ = 32,768 ms. The bin size sequence follows integer powers of 2 ($\left\{2^{i}|i\in \{0,1,2,...,15\}\right\}$), with a few intermediate values ($2^{6.5},2^{7.5},2^{8.5},2^{9.5}$). In order to evaluate the effect of binning methods, the activity within bins is determined by two distinct methods: the Presence of Spikes method and the Average Firing Rate method. Having the binned realizations, we infer the underlying model using MCMs framework. All the analyses are evaluated for four different populations of cells (n∈{10,15,20,30}). The general workflow followed in this paper is illustrated in Fig 1.

**Fig 1:**
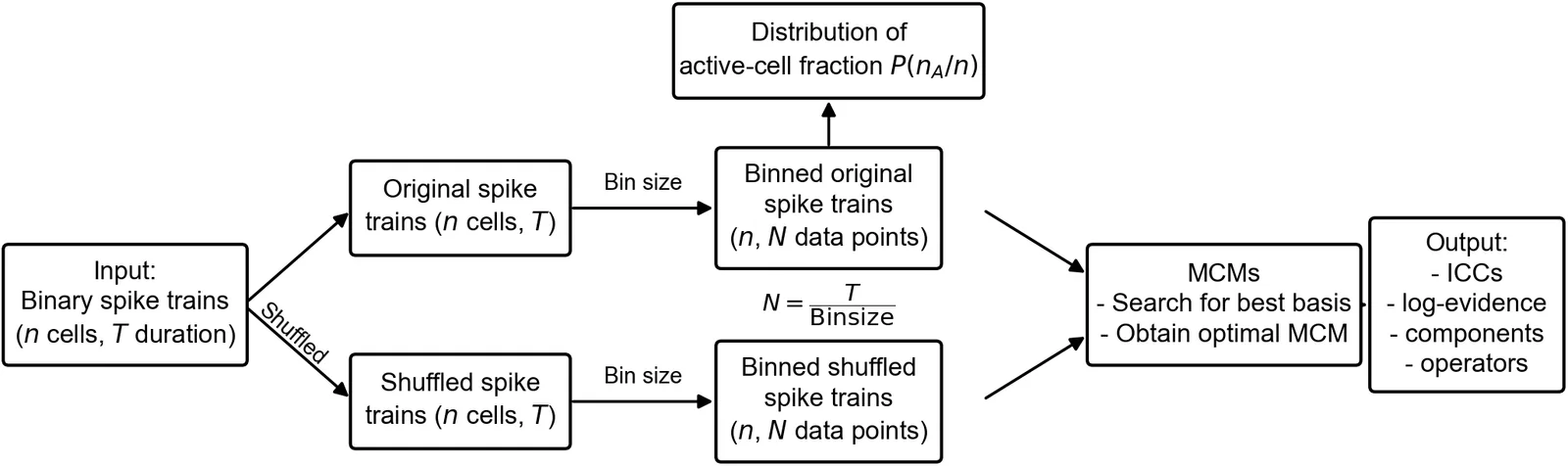
Workflow for processing spike trains belonging to a realization of a dataset. For each random realization (*n* cells, duration *T*), the binary spike trains are processed along both an original and a shuffled case. For each case, the spike trains are binned at a given bin size, resulting in *N* = *T*/(Bin size) data points, and the distribution of the active cell fraction $P(n_{A}/n)$ is computed from the original binned data. The binned original and shuffled data are each passed through the MCMs inference framework to obtain the best fitting model, from which the ICCs, log-evidence, and the components/operators of the inferred structure are extracted.

In the following, sample results are presented for one representative recording (**R1REMII**). The observed qualitative pattern identified here was consistent across the examined recordings. This cross-recording consistency is further validated quantitatively in this study, including a formal statistical agreement between the two informative resolution criteria across all 23 recordings, and a demonstration that the order richness and active cell fraction patterns generalize across the full data set (S2 Text). Representative cross-covariate comparisons are presented in S1 Text, and the complete set of results for all 23 recordings is openly available (see Data Availability Statement).

It is clear that the choice of temporal resolution can affect the observed activity pattern of cells in a data set. If we define the temporal resolution by the bin size used to bin a given binary data set, then the smallest value of bin size allows at most one event per bin, while the greatest value of the bin size captures all the activity of a cell by one single bin. For a 10-minute recording, the smallest and greatest bin sizes are 1 millisecond and 10 minutes (the full considered recording length), respectively.

Another important factor to emphasize is the binning procedure, as this factor can substantially affect the activity pattern of the cells within each bin. One can confirm that the method based on average firing rate implies stricter criteria in order to register a cell as active, and this potentially leads to sparser binned data than the method based on the presence of spikes. In any of these cases, binning the data affects the temporal resolution at which the data is analyzed, meaning that the decrease of the temporal resolution is equivalent to the increase of the bin size value. Consequently, the new spike patterns consist of unique information at each bin size value, which affects the inferred generative model.

In the following, we examine the key features of the inferred models, characterizing how the encoding of information depends on bin size, and how the prevalence of higher-order correlations depends on the temporal resolution at which the data is examined. By implementing the framework of MCMs, we obtained the independent complete components (ICCs) that describe the given binary data set and their associated operators through the heuristic basis-search and hierarchical-merging algorithm described in Materials and methods.

### ICCs

One can consider the number of ICCs as reflecting how compactly the structure of data is captured, since fewer, larger ICCs indicate that collective, higher-order correlations account for the data more efficiently than many small independent components. When considering the number of ICCs inferred from the realizations of the dataset, one can see that the average number of ICCs follows a curve with one clear global minimum around ($𝒪(10^{2})$  ms) and a slight flatness or local minima which takes place around ($𝒪(10^{4})$  ms) in the binning method based on average firing rate and ($𝒪(10^{3})$  ms) in the binning method based on the presence of spikes (Fig 2C and 2F). Evaluating the inferred models from both the original and the corresponding shuffled data shows that both have a global minimum: the global minimum of the original data occurs at around the same bin size as the global minimum of the difference plot (Fig 2A and 2D), while the global minimum of the shuffled data occurs at around the same bin size as the local minimum of the difference plot (Fig 2B and 2E). This can potentially tell us that results are sensitive to the number of spikes and the inferred correlation among cells shows limited data bias; otherwise, one might expect a straight line pointing at absolute independence of cells for all values of bin size (in the case of absolute independence, the number of ICCs and the number of cells are equivalent, in which each ICC contains one cell). One can observe this effect in shuffled data for small values of the bin size, where the correlation is absent and therefore, the number of ICCs on average is equivalent to the number of cells, while the lack of data points in coarse temporal resolutions leads to false correlation and consequently a smaller number of ICCs. A further notable observation across the original, shuffled, and difference cases is that the increase in the number of cells leads to fewer components up to the first global minimum, which can show us the importance of including more cells as this can reveal richer structure within the data.

**Fig 2:**
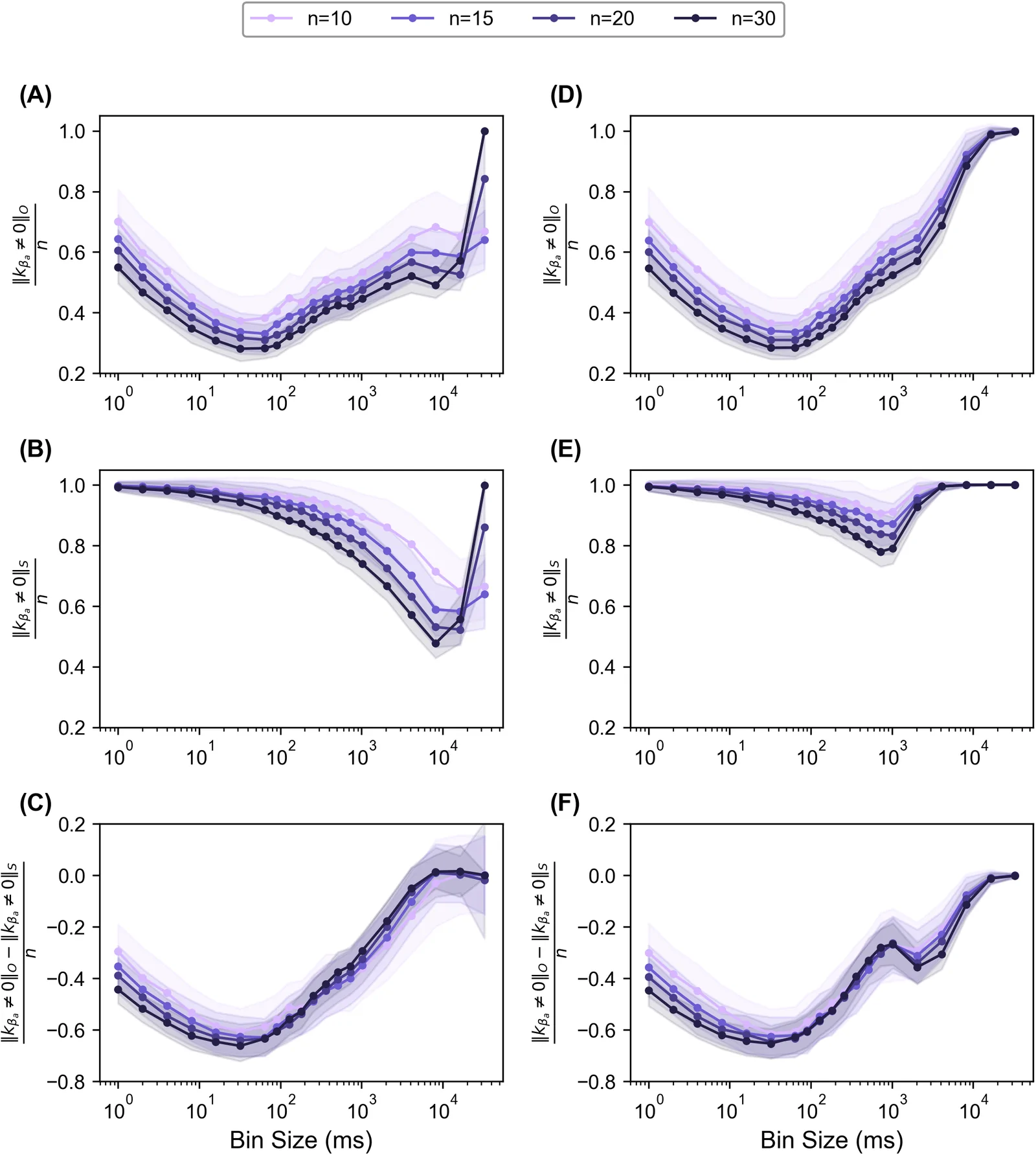
Normalized number of ICCs vs. bin size for R1REMII dataset. The plots are shown for two different methods of binning where the plots in the left column are based on the average firing rate and the ones in the right column are based on the presence of spikes. Plots for each binning procedure are shown for the original data set (panels A and D), shuffled data set (panels B and E) and the difference (panels C and F), where the difference of the number of the detected ICCs in the original data set with the corresponding shuffled version is considered. Shaded bands indicate ±1 standard deviation across the 200 realizations. All plots show a global minimum regardless of the number of cells and the binning method. Moreover, the greater the value of the cell’s population leads to fewer detected ICCs up to the global minimum.

### Log-evidence

By evaluating the log-evidence per data point of the detected models, one can confirm the same qualitative behavior (curves with minima) when considering the original and the corresponding shuffled cases, where the minima are centered around a value in $\left[10^{2},10^{4}\right]$ (Fig 3A, 3B, 3D and 3E).

**Fig 3:**
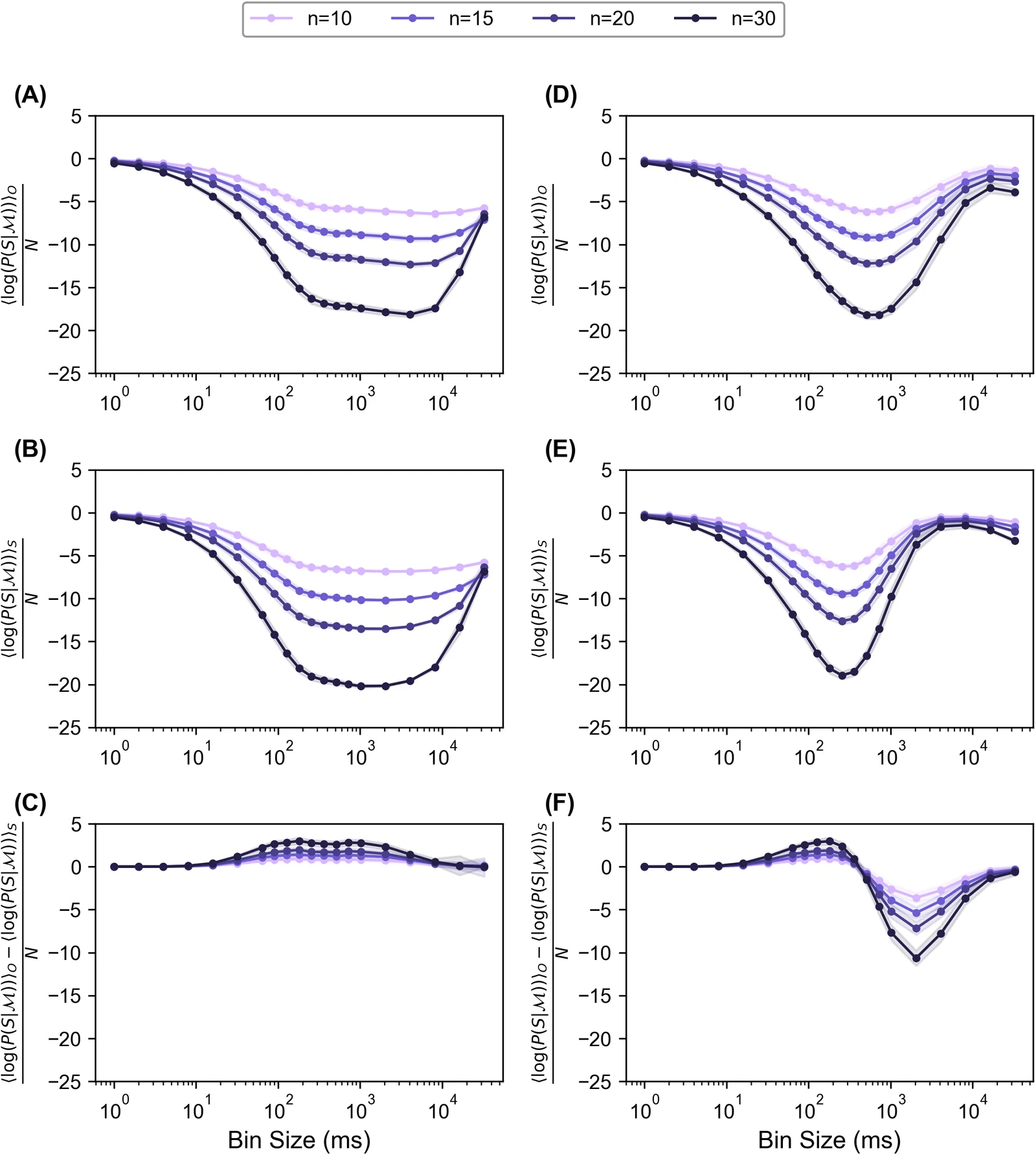
Log-evidence per data point vs. bin size for R1REMII dataset. The plots are shown for two different methods of binning where the plots in the left column are based on the average firing rate and the ones in the right column are based on the presence of spikes. Plots for each binning procedure are shown for the original data set (panels A and D), shuffled data set (panels B and E) and the difference (panels C and F), where the difference of the log-evidence per data point of the detected model in the original data set with the corresponding shuffled version is considered. Shaded bands indicate ±1 standard deviation across the 200 realizations.

However, one can get a clearer picture by evaluating the difference of the original and the corresponding shuffled case. These figures can potentially tell us for which range of bin size the log-evidence per data point is higher when avoiding the bias due to limited data. The difference plot shows the highest value of log-evidence per data point around $𝒪(10^{2})$  ms regardless of the binning method (Fig 3C and 3F). However, one can observe that around $𝒪(10^{3})$  ms the binning method based on the presence of spikes shows the poorest models according to log-evidence per data point by introducing false correlations due to a lack of data points. This false correlation increases as the number of cells increases, which leads to poorer models (Fig 3F). However, this effect appears in greater bin sizes in the binning method based on average firing rate, which can be seen by the gradual decrease of log-evidence after the highest value. Later on, we present the probability of active cells at different temporal resolutions, which can shed light on why this is observed.

In addition, if one attempts to normalize the log-evidence per data point curves by the number of cells ($\frac{{\langle \log(P(S|M)rangle}_{O}-{\langle \log(P(S|M))\rangle }_{S}}{N\times n}$), all the plots closely overlap, though not exactly (Fig 4). This can provide us a universal measure when it comes to the comparison of the models for different covariates, independent of the system size.

**Fig 4:**
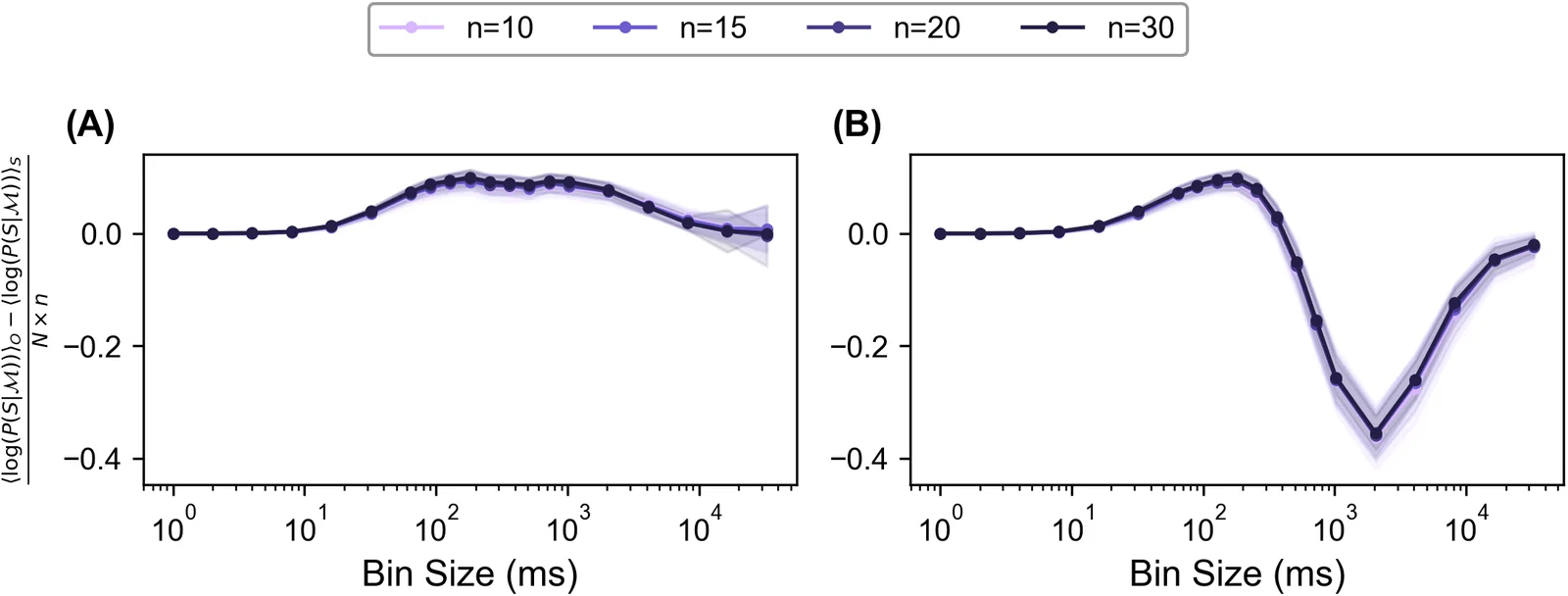
Normalized log-evidence per data point vs. bin size when considering the difference between the original R1REMII dataset with the corresponding shuffled version. The plots are shown for two different methods of binning based on (A) the average firing rate and (B) the presence of spikes. In both, the curves are normalized by the value of the *n*. Shaded bands indicate ±1 standard deviation across the 200 realizations. This shows a near-universal behavior regardless of the cell population size that is considered in the analysis.

To this point, the results provide a good overview of how efficiently information is encoded by a minimal number of variables consistent across different population sizes at around $𝒪(10^{2})$  ms, and the goodness of the inferred model around this value in comparison to other temporal resolutions.

We can see that the bin size at which the shuffle-corrected log-evidence per data point (Fig 4) is maximal is consistent with the point where the shuffle-corrected ICC count (Fig 2) is most consistent across different population sizes. For R1REMII data set, these two points nearly agree with each other, especially when using binning method based on average firing rate.

### Orders of interaction

According to the results of ICCs, having fewer components than the number of cells suggests the presence of multiple cells in each component. In order to visualize this more clearly, we examine how the number of cells in components/operators is affected by the temporal resolution. To do so, we followed the procedure mentioned in Orders of interaction for both components and operators inferred by MCMs procedure. The number of cells that are uniquely present in components/operators can be regarded as the order of interaction. For instance, if a component/operator is a product of three cells, then this component/operator is of the third order of interaction.

The difference heat-maps show both the temporal resolutions at which the higher orders of interaction become prominent and the resolutions at which bias introduces spurious interactions of any order. For both binning methods, results show that around $𝒪(10^{2})$  ms, we can observe the presence of the higher orders of interaction where there are components/operators that include greater fraction of the population of the cells. However, a similar pattern also appears at large bin sizes, where lack of data points can inflate false correlations among cells and produce higher-order interactions driven by bias (ocher shades in Fig 5).

**Fig 5:**
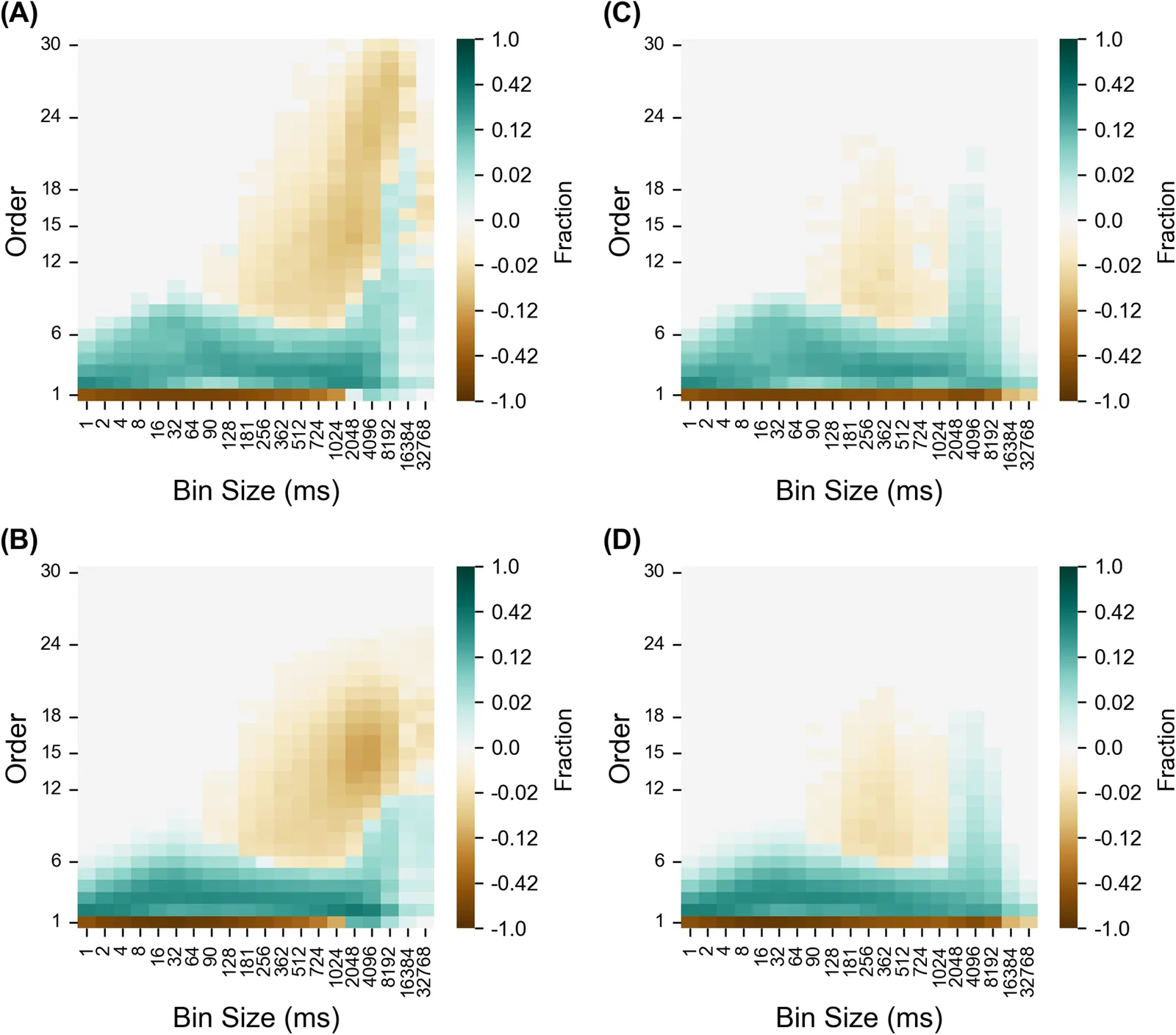
Fraction of components (panels A and C) / operators (panels B and D) that contain *n* cells (Order *n*), presented for the R1REMII grid cell dataset, *n* = 30 cells. The plots are shown for two different methods of binning where the plots in the left column are based on the average firing rate and the ones in the right column are based on the presence of spikes. Values are visualized in re-scaled format, while preserving the original values on the color bar.

However, this shows a difference between the two binning methods: the biased region which again starts around $𝒪(10^{2})$  ms in both methods (ocher shades in Fig 5), is more pronounced for the average firing rate method, extending to higher orders and a wider range of bin sizes. This is consistent with the easier criterion applied to presence of spike method when binning the data, which leads to more saturated activity at large bin sizes (next section), and consequently little residual variability across cells for spurious correlations to exploit. On the other hand, the method based on average firing rate retains substantial variability even at larger bin sizes, and therefore is more susceptible to spurious higher-order correlations due to the sampling bias.

### Distribution of active cells across different bin sizes

In order to have a general overview of the effect of temporal resolution on the activity of the cells, we examine the distribution of active cells across different bin sizes. As mentioned before, this analysis was done on the original data set, and we solely depend on activity status of cells regardless of the frequency of their activity within each bin. Results reveal several notable patterns, including a mean fraction of active cells per bin size. This quantity follows a sigmoid-like pattern which is dependent on the binning method; the sigmoid-like pattern has saturation at different values of the fraction for active cells, in which by the saturation we mean the value at the largest bin sizes considered in this paper. It is expected that if the bin size equals the full length of the recording, both binning procedures result in the same value, which shows the activity of the cell by a single number. Across the tested range of bin sizes, the binning method based on the average firing rate leads to a sparser representation than the other method (saturating at ~44% compared to ~100%) (Fig 6). In both methods, the sigmoid-like pattern shows almost the same behavior up to bin size around $b_{div}^{*}$. After this point, the mean active fraction diverges between the two binning methods. Furthermore, the general trend of the mean active fraction is consistent across datasets and across the different population sizes considered in the study (S4 Fig).

**Fig 6:**
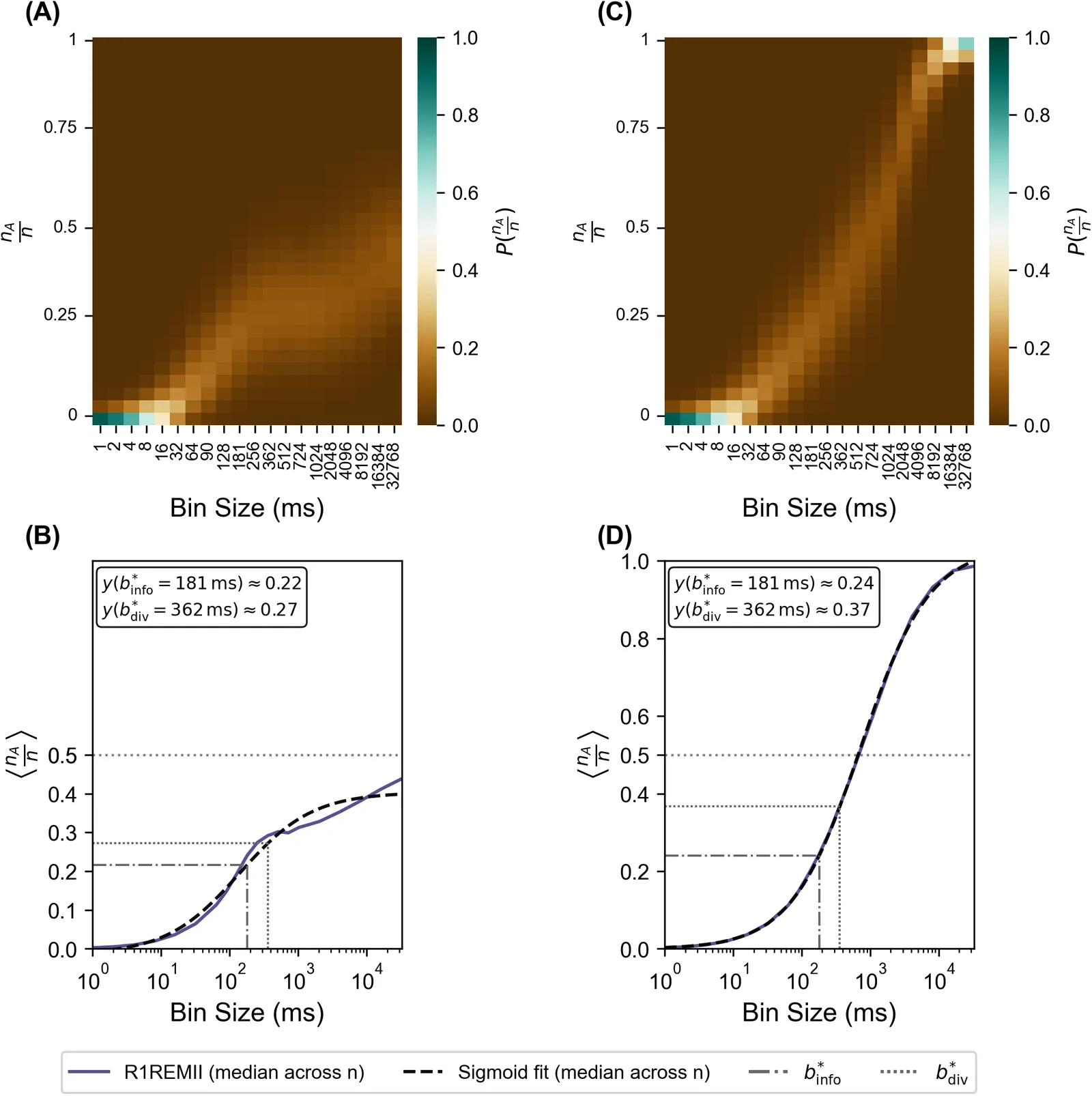
Active cell fraction vs. bin size in the R1REMII recording. The heat maps (A, C; *n* = 30) show the probability of each active cell fraction after binning the data at each bin size, and the line plots (B, D) show the median, across population sizes n∈{10,15,20,30}, of the mean active cell fraction vs. bin size, along with a sigmoid fit. The plots are shown for two different methods of binning where the plots in the left column (A, B) are based on the average firing rate and the ones in the right column (C, D) are based on the presence of spikes. The mean active cell fraction follows a sigmoid-like pattern where the saturation takes place around (A and B) ~44% and (C and D) ~100% of the total cells. In the line plots (B, D), $b_{info}^{*}$ and $b_{div}^{*}$ are indicated with boxes reporting the active cell fraction at each point.

In each method, where the mean active cell fraction approaches half of the saturated fraction marks a natural transition point between sparse and distributed representations, and therefore plays an important role in determining the inferred interaction structure. To be more precise, where the binning procedure is done based on the presence of a spike in bins, saturation takes place when almost 100% of cells are active, while when considering the binning method based on average firing rate, the saturation takes place when almost half of the cells are active (~44% for R1REMII), which in both methods the saturation takes place on the order of $𝒪(10^{4})$ milliseconds. For the presence of spikes method, the ~50% active fraction marks the mid-point of the sigmoid-like pattern. One can confirm the symmetrical distribution of the fraction of active cells at this point, while smaller (larger) bin sizes lead to a bell-shaped distribution centered around small (large) number of cells, signaling the most frequent fraction (Fig 7).

**Fig 7:**
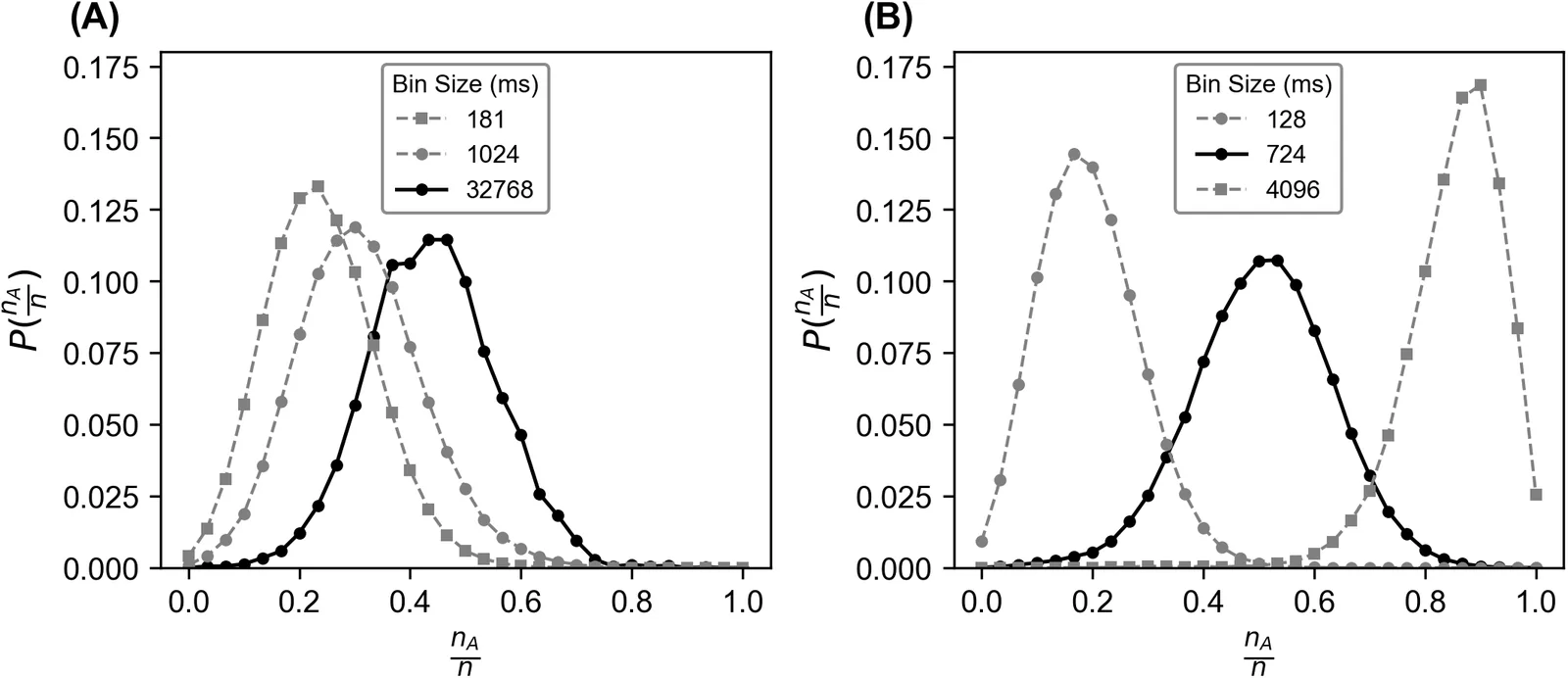
Distribution of the active cell fraction at representative bin sizes. The $P(n_{A}/n)$ distribution for the R1REMII recording (*n* = 30) is shown at representative bin sizes, for the binning method based on (A) average firing rate and (B) the presence of spikes. The distribution is shown for three representative bin sizes: the bin size at which the peak of the distribution lies closest to half of the cells being active (solid black line), and two other bin sizes for comparison (dashed gray lines). In both panels (A, B), the distribution is centered around a small number of active cells at small bin sizes and shifts toward a larger number of active cells at large bin sizes.

Based on the results across recordings, at the bin size where two methods of binning start to deviate when considering the distribution of the active cells ($b_{div}^{*}$), the distributions are centered around ~27% for binning based on average firing rate and 34% for binning based on presence of spike, a less sparse regime than the informative bin with ~18% of active cells (S5 Fig). Potentially, this finding can have broader implications for identifying informative sparsity levels in neural systems.

### Cross-recording consistency

As mentioned earlier, the qualitative results are consistent across recordings, although the quantitative details vary across recordings, animals, and behavioral states. In this section, we are presented with analysis across recordings. Therefore, we address the informative bin size for each recording by considering the crossover bin size $b_{\sigma (b)}^{*}$ and the bin size where normalized log-evidence is maximal $b_{\tilde{\ell }}^{*}$. For the representative recording R1REMII, these two points approximately agree with each other (Fig 8). Note that $b_{\sigma (b)}^{*}$ is obtained based on a search within a range around the minimum of the median plot for normalized ICCs. The reason for considering a range around the minimum and not the full bin sizes is to avoid the low dispersion that takes place around the shallower dip of the normalized ICCs plot particularly for the binning method based on presence of spikes (Fig 2F).

**Fig 8:**
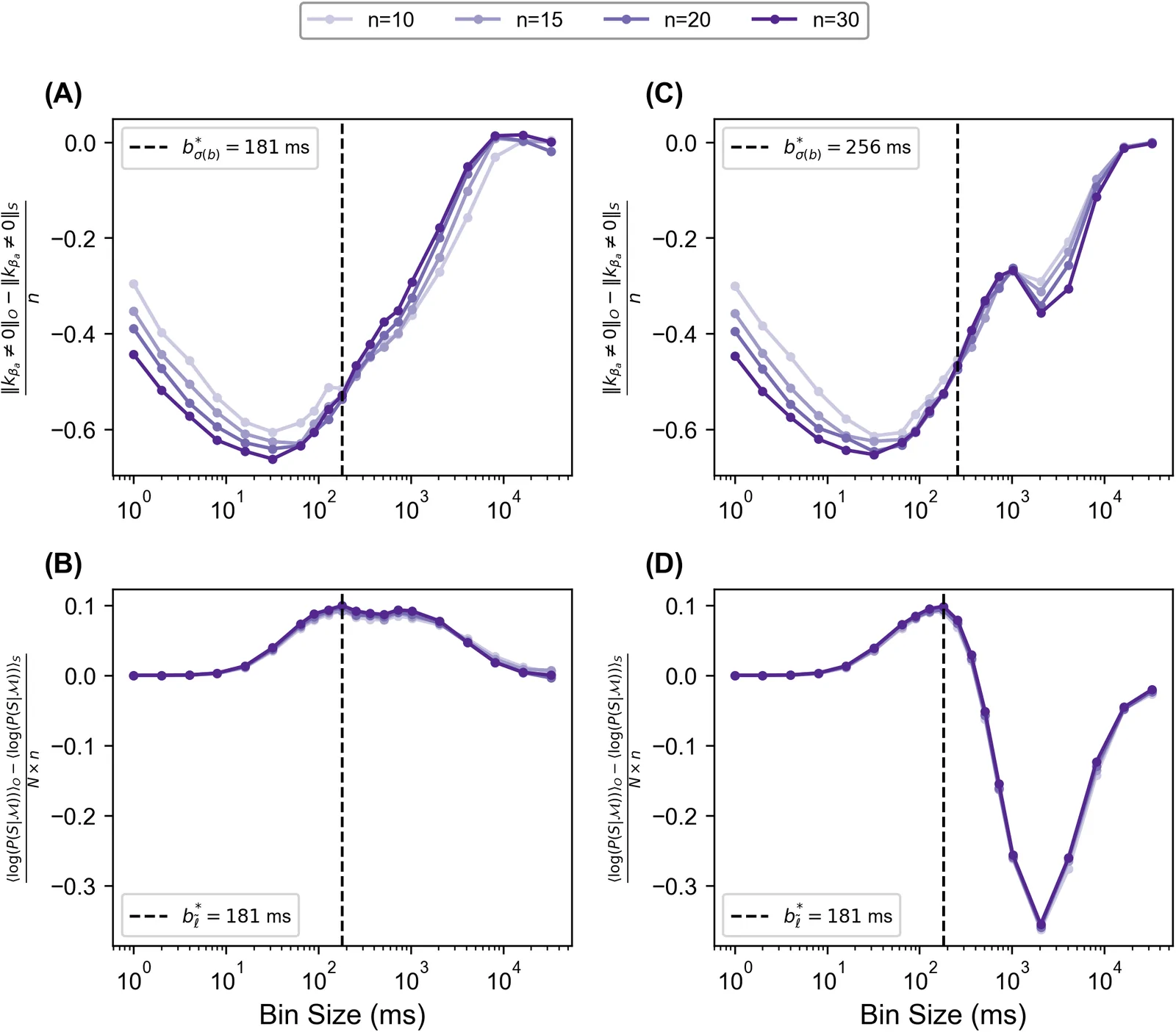
Normalized ICC and log-evidence considering the difference between the original dataset with the corresponding shuffled version, for R1REMII recording. Normalized ICCs count vs. bin size for different values of *n* is plotted for binning methods based on (A) average firing rate and (C) presence of spikes. In both plots we notice that there exists an intermediate bin size (crossover bin $b_{\sigma (b)}^{*}$) at which the curves for different values of *n* have minimal distance to each other. The subsequent normalized log-evidence vs. bin size is shown for the same values of *n* for two binning methods based on (B) average firing rate and (D) presence of spikes. The peak bin size for normalized log-evidence ($b_{\tilde{\ell }}^{*}$) along with the crossover bin size are indicated by dashed lines. The results for this specific recording show the close agreement between the two criteria discussed throughout the paper.

A natural question that arises is whether or not this observation is consistent among all recordings. Having a pair $(b_{\sigma (b)}^{*},b_{\tilde{\ell }}^{*}{)}_{i}$ for each recording *i*, we can make the scatter plot in which the diagonal line represents the exact agreement where $b_{\sigma (b)}^{*}=b_{\tilde{\ell }}^{*}$(Fig 9). Based on the same pair of values, the histogram of $\Delta_{i}=\log_{2}(b_{\tilde{\ell },i}^{*}/b_{\sigma (b),i}^{*})$ shows the distribution of the log-distance from the diagonal line (Fig 9). The results show that, for both binning methods, 96% of the recordings lie within a factor of two of exact agreement ($|\Delta_{i}|\leq 1$), with $b_{\sigma (b)}^{*}$ and $b_{\tilde{\ell }}^{*}$ having a median of 181 ms across both binning methods. The values of both criteria along with $b_{info}^{*}$ and $b_{div}^{*}$ are reported in Table 3.

**Fig 9:**
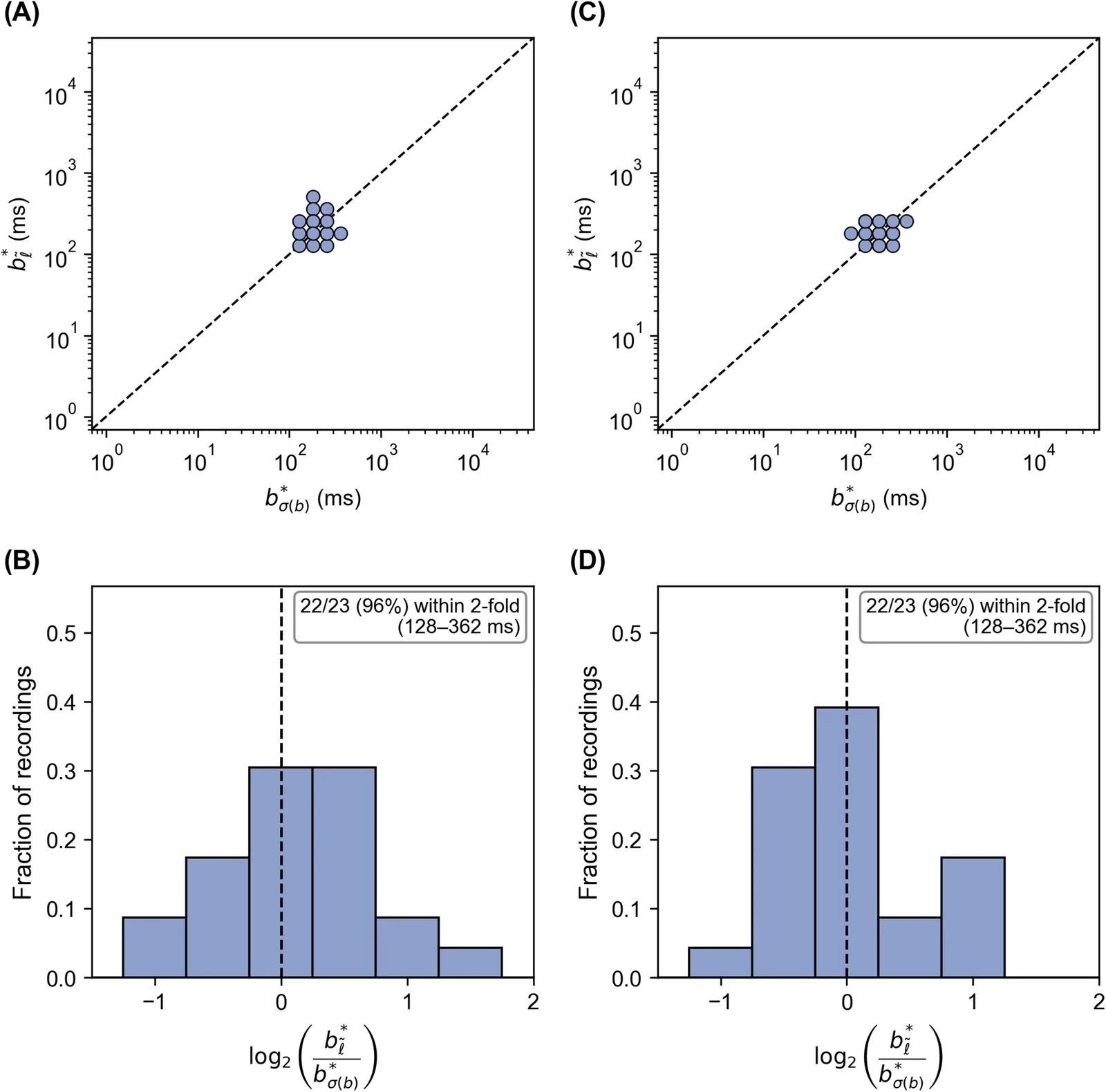
Cross-recording agreement between the normalized ICC crossover bin and the normalized log-evidence peak bin size. The relation between $(b_{\sigma (b)}^{*},b_{\tilde{\ell }}^{*}{)}_{i}$, for each recording *i* from 23 recordings is evaluated considering two binning methods: (A, B) average firing rate and (C, D) presence of spikes. Top row (A, C) demonstrates the scatter plot of $(b_{\sigma (b)}^{*},b_{\tilde{\ell }}^{*}{)}_{i}$ across all recordings. The diagonal line $b_{\sigma (b)}^{*}=b_{\tilde{\ell }}^{*}$ indicates the exact agreement. Bottom panels (B, D) are histograms of $\Delta_{i}=\log_{2}(b_{\tilde{\ell },i}^{*}/b_{\sigma (b),i}^{*})$ for the same recordings as the top panels. The dashed line at 0 is equivalent to the diagonal line when both crossover and peak bin size indicate the same value.

**Table 3:** Informative and divergence bin sizes (ms) for all recordings. $b_{\sigma (b)}^{*}$: ICC/n crossover bin. $b_{\tilde{\ell }}^{*}$: LogEv/n peak bin. $b_{info}^{*}$: geometric mean of the two (Materials and methods). $b_{div}^{*}$: bin size at which the two binning methods’ active cell fraction curves diverge (Materials and methods).

Recording	Average Firing Rate			Presence of Spikes			$b_{div}^{*}$
	$b_{\sigma (b)}^{*}$	$b_{\tilde{\ell }}^{*}$	$b_{info}^{*}$	$b_{\sigma (b)}^{*}$	$b_{\tilde{\ell }}^{*}$	$b_{info}^{*}$	
R1OFI	128	128	128	128	128	128	256
R2OFI	256	128	181	181	128	128	362
R3OFI	181	181	181	256	128	181	362
R1WWI	128	181	128	181	181	181	362
R2WWI	181	181	181	181	181	181	512
R3WWI	256	181	181	181	181	181	512
R1OFII	181	256	181	181	181	181	512
R2OFII	181	256	181	128	256	181	724
R3OFII	256	362	256	362	256	256	724
R1REMII	181	181	181	256	181	181	362
R2REMII	256	181	181	256	181	181	362
R3REMII	256	181	181	256	181	181	512
R1SWSII	362	181	256	181	181	181	512
R2SWSII	128	256	181	128	256	181	724
R3SWSII	181	256	181	181	181	181	724
Q1OF	181	512	256	181	181	181	362
Q2OF	181	256	181	256	256	256	512
Q1WW	181	362	256	128	181	128	512
Q2WW	256	256	256	181	256	181	724
Q1SWS	181	181	181	90	181	128	512
Q2SWS	181	256	181	128	256	181	512
Q1REM	181	128	128	181	128	128	256
Q2REM	181	181	181	181	128	128	362
**Median [IQR]**	**181 [181, 256]**	**181 [181, 256]**	**181 [181, 181]**	**181 [154, 218]**	**181 [181, 218]**	**181 [154, 181]**	**512 [362, 512]**

Beyond the location of the informative bin size, the qualitative features that characterize it for the R1REMII example, i.e., rich higher-order structure (Fig 5) and sparse active cell representation (Figs 6 and 7), generalize across all 23 recordings and both binning methods (see S2 Text and S3 and S4 Figs).

## Discussion

### Inferring interactions without a predefined model

Identifying the higher-order interactions present in data recorded from complex interacting systems has always been one of the central concerns. Traditional models attempt to infer these interactions by pre-defined models, which prohibit the evaluation of the potential orders of interaction that are absent in the defined model. However, our knowledge of the underlying generative system is often limited, for instance, it is not clear exactly how the brain puts together the information from several neurons [35], and making any assumption about this can potentially lead to false interpretations, with no actual guarantee that the imposed assumption is able to capture all the information present in the data. The story gets more complicated when comparing the importance of the higher-order interactions across different covariates. In order to have a meaningful comparison, first and foremost, one must account for the amount of present information in the data. One important underlying variable that determines the amount of information present in data is the temporal resolution at which the data is studied. Clearly, at fine temporal resolutions, all the fine dynamical responses of the system's elements are captured, while this variability is summarized into a single response at coarser temporal resolutions. Either of these choices substantially reshapes the correlation structure in the data and, consequently, the information that one can obtain from the data [36]. This important factor has been studied extensively when it comes to extracting information from the recording of individual system elements [37–45], but is not systematically investigated at the population level, where the activity of every individual cell is not isolated from other cells and the external stimulus is not the only factor shaping how the neuron responds. This paper demonstrates the key role of the temporal resolution in the encoding of information and the relevance of the higher orders of interaction. Based on the inference method used in this study, one can systematically infer all the present interactions directly from the data without any prior assumption on the interactions of the underlying model. This inference provides a good overview of the number of components needed in order to encode the information efficiently and the orders of interactions involved, and shows that these two factors are highly affected by the temporal resolution at which the data is studied. It must be emphasized that the inferred interaction structure in this context reflects statistical dependencies among recorded units, not functional dependencies, causal influences, or biological connectivity among units.

### Two criteria for the informative temporal resolution

In order to identify the informative temporal resolution, two points are considered throughout this study: the bin size at which the number of independent complete components (ICCs) is minimal and at the same time least affected by the population size, and the bin size at which the normalized log-evidence per data point for the inferred model is maximal. In practice, both these bins answer one question from different angles: How compact the data can be encoded at a given temporal resolution. $b_{\sigma (b)}^{*}$ and $b_{\tilde{\ell }}^{*}$ defined in this study are best understood as representative temporal resolutions within a narrow window at which both criteria are satisfied (Materials and methods). Therefore it is more accurate to state that the informative region for each considered recording within this study is within a narrow region of the $𝒪(10^{2})$ ms, and not a single point. The normalized log-evidence is however a more robust anchor. It is natural to think that the resolution at which the ICC count is minimal, is the most efficient one. *However, this minimum depends on the amount of data and on the population size: since the number of possible activity patterns grows exponentially with n, it carries a finite-sampling bias that grows with n* [46,47]. On the other hand, the normalized log-evidence calculation already takes into account the uncertainty over parameters, which explains the collapse of curves for different population sizes. For the same reasoning, it is more meaningful to consider the minimum number of ICCs when this value is minimal and at the same time where the curves for different population sizes converge.

### Orders of interaction depend on temporal resolution

Temporal resolution must also be taken into account when it comes to the comparison of the importance of the orders of interaction. For instance, one cannot simply claim that “second order of interaction is always prominent”; rather, one can state that “second order interaction is prominent when the data is considered at the finest scale”. In our case study (R1REMII), when considering components, the second order of interaction is more prominent than the $6^{th}$ order when the data is studied at 1 *ms*. However, around $𝒪(10^{2})$ ms this prominence is reversed. This can be confirmed in the second and sixth rows of the heat-maps in Fig 5 at the specified temporal resolution or bin size in milliseconds.

### Two pivotal temporal resolutions

For grid cells, this study identified two temporal resolutions as the most important. One occurs at intermediate resolution ($𝒪(10^{2})$ ms), where the encoding complexity is minimal and independent of population size. This point corresponds to a minimal number of independent components, maximal normalized log-evidence per data point, rich correlational structure, and a sparse representation of the network. Another occurs at coarse resolution, where the representation becomes distributed. Note that this point is also where false correlations are most dominant due to the bias coming from the limited data (ocher region in Fig 5) and corresponds to minimal number of ICCs and minimal log-evidence when encoding the shuffled data. Although distributed representations show robustness and high capacity in storing and retrieving information [48], they are harder to infer reliably from limited data, as observed at coarse resolutions in this study. This contrast can be observed where higher orders of interaction are present (Fig 5) despite the poor inferred model (Fig 4) (note that the minima of the log-evidence plots happen around the time resolutions where distributed representation occurs). The sparse and intermediate resolution regime is also consistent with efficient representation of the population: at this resolution, the data is explained by the fewest effective independent components that explain the population's joint activity. This is validated by the normalized log-evidence which balances the model fit against representational cost. As per Shannon's idea, the compression of the data is best done when the representational cost is minimal [49,50]. This study shows that for grid cells data, there exists a temporal resolution at which the trade-off between model fit and the representational cost is optimized, and consequently the effective dimensionality of the population code is minimized.

### Temporal resolution and comparisons across co-variates

Since both the amount of information and order of interaction that appear to be dominant are strongly affected by temporal resolution, when comparing the importance of orders of interaction across different covariates, behavioral states, experimental conditions, or otherwise, one must first and foremost account for temporal resolution. Temporal resolution therefore must be a fixed methodologically defined reference prior to interpreting differences between covariates, as the results might not be necessarily consistent across different temporal resolutions (see S1 Text and S1 and S2 Figs).

### Limitations

Some points worth reiterating and having in mind when going through this study. First and foremost, the interaction structure inferred here reflects statistical dependencies among recorded units under MCMs framework, and does not reflect functional or causal relationships, or biological connectivity. The informative temporal resolution identified in this study is more accurately defined as a narrow region defined by two considered criteria; its numerical location is specific to the present data set and binning methods, and should be re-derived, using the same criteria or logic, for other recordings. When considering the active cell fraction, the quantified divergence behavior of the binning methods considered in this study takes place at coarser temporal resolutions, as a result of the binning procedure properties. Finally, the numerical validation is performed over 23 recordings from grid cells. Extending this framework to other neural populations is a natural direction for future work.

The framework used in this study can be applied when studying complex interacting systems, particularly when the available data is limited: one can first determine the most informative temporal resolutions for a given data set, and then continue with further analysis in an accurate and systematic setting.

## Conclusion

This study sheds light on the importance of temporal resolution in studying complex interacting systems based on limited datasets. By employing the framework of the Minimally Complex Models, we systematically inferred the statistical interaction structure of complex interacting systems, with no prior assumptions about the structure across different temporal resolutions.

As stated in Discussion above, the amount of information in data, the relevance of different orders of interaction, and the validity of comparison across covariates are all dependent on the temporal resolution at which the data is studied.

In conclusion, this work provides a systematic approach in order to understand the underlying pattern of generative model in complex interacting systems based on limited data, with implications in different fields such as neuroscience and beyond.

## Supporting information

S1 Text Temporal resolution affects the comparison of the significance of the orders of interactions. Additional details are provided in the file.
(PDF)

S1 Fig Comparison of the different experimental co-variates for each module of rat R on day II, considering the order of interaction in components, at bin sizes of 1 and 128 milliseconds. Each row corresponds to one of rat R's three modules (R1–R3). Within each panel, box plots show the shuffle-corrected probability of each component order (Order 1–6), colored by experimental state (OF, REM, SWS). Columns 1–4 (panels A–C, D–F, G–I, J–L) show, respectively: average firing rate at 1 ms, average firing rate at 128 ms, presence of spikes at 1 ms, and presence of spikes at 128 ms.
(TIF)

S2 Fig Comparison of the different experimental co-variates for each module of rat R on day II, considering the order of interaction in operators, at bin sizes of 1 and 128 milliseconds. Each row corresponds to one of rat R's three modules (R1–R3). Within each panel, box plots show the shuffle-corrected probability of each operator order (Order 1–6), colored by experimental state (OF, REM, SWS). Columns 1–4 (panels A–C, D–F, G–I, J–L) show, respectively: average firing rate at 1 ms, average firing rate at 128 ms, presence of spikes at 1 ms, and presence of spikes at 128 ms.
(TIF)

S2 Text Order richness, active cell fraction, and their divergence bin generalize across all 23 recordings. Additional details are provided in the file.
(PDF)

S3 FigOrder richness across all 23 recordings.Δ⟨i⟩(b) (S2 Text) for components (top row) and operators (bottom row), based on the average firing rate (left column) and presence of spikes (right column) binning methods. In each panel, thin lines show individual recordings; the thick line and shaded band show the median and interquartile range across recordings; the vertical dashed line and shaded band mark the median (and interquartile range) for the corresponding method's informative bin $b_{info}^{*}$. The horizontal dotted line marks Δ⟨i⟩=0.(TIF)

S4 FigActive cell fraction across all 23 recordings.$\langle n_{A}/n\rangle (b)$ (Materials and methods) based on (A) the average firing rate and (B) the presence of spikes binning methods. Thin lines show the sigmoid fit for each individual recording (Fig 6), the thick line and shaded band show the median and interquartile range across recordings, the vertical dashed line and shaded band mark the median (and interquartile range) of $b_{info}^{*}$, and the dotted vertical line and shaded band mark the median (and interquartile range) of $b_{div}^{*}$ (Materials and methods). Horizontal reference lines mark $n_{A}/n=0.5$, the median active cell fraction at $b_{info}^{*}$, and the median active cell fraction at $b_{div}^{*}$ for that panel's binning method.(TIF)

S5 FigRelationship between the informative bin and the divergence bin.$b_{info}^{*}$ compared to $b_{div}^{*}$ (Materials and methods) across all 23 recordings, based on (A, B) the average firing rate and (C, D) the presence of spikes binning methods. Each thin line connects one recording's $b_{info}^{*}$ to its $b_{div}^{*}$; the thick black line marks the median. (A, C) show the $\log_{2}$ shift in bin size, $\log_{2}(b_{div}^{*}/b_{info}^{*})$; (B, D) show the corresponding shift in active cell fraction $n_{A}/n$.(TIF)
