# Supplementary material for: Temporal resolution shapes inferred higher-order interactions and information representation in neural population activity: A data-driven approach using minimally complex models
Source: PLoS One. 2026 Oct 26;21(10):e0322347. doi: 10.1371/journal.pone.0322347 (PMC13705606; doi:10.1371/journal.pone.0322347)
Supplement: Supplementary file 1 [file pone.0322347.s001.pdf]

S1 Text. Temporal resolution affects the comparison of the significance of the orders of interactions

In this part, the dependency of the higher orders of interaction on external and internal co-variates is addressed. Based on the results, there is no absolute statement that one can make about the prominence of orders of interaction in different experimental states or modules. This prominence is highly dependent on the temporal resolution at which each order of interaction is studied. Having the temporal resolution as a fixed parameter, one can attempt to compare different co-variates. For instance, for rat R on day II, considering temporal resolutions $\in \{1, 128\}$ milliseconds for both binning methods, we can see patterns that are unique to these resolutions. If we associate the difference of the order's probability ($P(Order)_O - P(Order)_S$) as significance, when considering 1 millisecond temporal resolution, REM shows lower significance for single cell operators than OF and SWS, while for operators with three cells, REM has the highest significance (S2 Fig). On the other hand, on the scale of 128 milliseconds, operators with first and second orders show the highest significance for REM, followed by slightly more significance of the OF than SWS, while this pattern is reversed for operators of orders 3–5 (S2 Fig); at order 6, the highest and smallest-magnitude order, this reversal is less consistent across modules. These results for orders 1–5 are consistent in both binning methods. When considering the component's order, one can see the dependence of the judgment on the temporal resolution (S1 Fig). In general, results confirm the important role of the temporal resolution on the significance of the co-variates for different orders of interaction.
