# Supplementary material for: Temporal resolution shapes inferred higher-order interactions and information representation in neural population activity: A data-driven approach using minimally complex models
Source: PLoS One. 2026 Oct 26;21(10):e0322347. doi: 10.1371/journal.pone.0322347 (PMC13705606; doi:10.1371/journal.pone.0322347)
Supplement: Supplementary file 2 [file pone.0322347.s004.pdf]

S2 Text. Order richness, active cell fraction, and their divergence bin generalize across all 23 recordings

In the main text, two features of the informative bin size were discussed for a single representative recording (R1REMII): the dominance of higher-order interactions in the inferred components and operators (Fig 5), and a sparse active cell representation of the population (Figs 6 and Fig 7). As it is shown in Fig 9, the crossover bin $b^*_{\sigma(b)}$ and the log-evidence peak bin $b^*_{\bar{\ell}}$ almost agree with each other. In addition to this, we evaluate the behavior of the two binning methods as the bin sizes increase, especially at a point systematically related to $b^*_{\rm info}$. In this section, we show through statistical analysis that the dominance of higher order interactions, the proportion of active cells, and the relationship between the informative bin and the divergence point all behave consistently across 23 recordings.

Orders of interaction. In order to address this across 23 recordings, we define the order richness at bin size b as:

$$\Delta\langle i\rangle(b) \;\equiv\; \langle i\rangle_O(b) - \langle i\rangle_S(b), \qquad \langle i\rangle(b) \;=\; \sum_{i=1}^{n} i \cdot P(\text{order} = i \mid b),$$

where the subscripts O/S denote the original and shuffled data respectively (see Materials and methods), and $P(\text{order} = i \mid b)$ is the probability that a detected component (or operator) at bin size b contains i cells. Therefore, $\Delta\langle i\rangle(b)$ is the shuffle-corrected mean order of the inferred structure at bin size b. Clearly, the large value of $\Delta\langle i\rangle(b)$ indicates that on average higher-order components/operators are detected. S3 Fig shows the median curve (bold line) and interquartile range (shaded band) across all 23 recordings, along with individual recordings shown by thin lines. We can see that the order richness increases from the finest bin size and shows a peak close to $b^*_{\rm info}$ regardless of the binning methods for both components and operators. This result confirms that the informative bin coincides with where higher-order structure is most pronounced. However, two binning methods diverge in behavior right after the informative bin size. For the case based on average firing rate, the order richness sharply falls until partial recovery consistent with the limited-data bias already described for this method at coarse resolutions. For the case based on presence of spike, it falls into a shallower minimum before rising to a secondary peak which can be explained by the near saturated activity mentioned for this method at coarse bin sizes.

Active-cell fraction. Similarly, we evaluate the active-cell fraction $\langle n_A/n\rangle$ for each recording at its own informative bin (S4 Fig). At the informative bin, the median active-cell fraction across 23 recordings is 18.2% (IQR 16.5–19.6%) for the average firing rate method and 17.5% (IQR 15.3–20.2%) for the presence of spikes method. This value agrees closely for two binning methods for all recordings (see Table A). This indicates that the sparse regime at the informative bin is not an artifact of the choice of binning method.

Divergence bin. In order to understand the behavior of the active cell fraction across two binning methods, we ask whether the behavior of the two binning methods diverge at a bin size systematically related to the recording's own informative bin. We define the divergence bin $b^*_{\rm div}$ as the smallest bin size at which the two methods' active cell fraction curves differ by at least 5 percentage points (see Materials and methods). The result across the 23 recordings show a correlation between $b^*_{\rm div}$ and $b^*_{\rm info}$ for both binning methods (Pearson $r = 0.543$, $p = 0.0074$ for average firing rate; $r = 0.513$, $p = 0.0123$ for presence of spikes), with $b^*_{\rm div}$ occurring after $b^*_{\rm info}$ in all recordings. In both binning methods, the median $\log_2(b^*_{\rm div}/b^*_{\rm info})$ is $+1.50$, which means that the divergence bin is on average $\sim$2.8-fold larger than the informative bin (S5 Fig A, C). Consequently, the active-cell fraction is higher at the divergence point, rising from a median of 18.2% to 27.3% for the average firing rate method and from 17.5% to 34.0% for the presence-of-spikes method (S5 Fig B, D).

Together, the results across 23 recordings confirm that the presented results for R1REMII generalize across the full set of recordings: regardless of the binning method, around the informative bin size, the higher order interactions are dominant, the active population remains sparse there ($\sim$ 18%), and the two binning methods diverge slightly after this point at a bin size that scales consistently with each recording's own informative bin.

**Recording**	**Average firing rate (%)**	**Presence of spike (%)**	**Difference (%)**
R1OFI	18.66	20.06	+1.4
R2OFI	19.68	16.86	−2.8
R3OFI	19.58	20.87	+1.3
R1WWI	14.80	18.54	+3.7
R2WWI	17.27	17.50	+0.2
R3WWI	16.93	16.85	−0.1
R1OFII	16.64	17.75	+1.1
R2OFII	13.85	13.26	−0.6
R3OFII	15.79	16.27	+0.5
R1REMII	21.63	23.99	+2.4
R2REMII	19.59	21.68	+2.1
R3REMII	19.35	20.34	+1.0
R1SWSII	21.37	17.12	−4.2
R2SWSII	16.09	14.87	−1.2
R3SWSII	15.73	14.68	−1.1
Q1OF	19.87	18.94	−0.9
Q2OF	16.41	20.92	+4.5
Q1WW	18.62	13.84	−4.8
Q2WW	17.88	14.23	−3.7
Q1SWS	18.19	13.97	−4.2
Q2SWS	16.67	15.74	−0.9
Q1REM	19.59	21.99	+2.4
Q2REM	20.71	18.13	−2.6

Table A: **Active cell fraction at the informative bin, per recording and binning method.** For each recording, the active-cell fraction $\langle n_A/n \rangle$ is reported at that recording's own informative bin b^*_{info}, for each binning method, along with their difference. All 23 recordings agree within 5%.
